# Continuous Mass Versus Layered Fascial Closure in Contaminated Emergency Midline Laparotomies: A Prospective Observational Cohort Study

**DOI:** 10.7759/cureus.115417

**Published:** 2026-08-29

**Authors:** Arunav Roy, Hitesh Kumar, Dipendra K Sinha, Somya Verma, Ritvik Prakash, Supriya Sahoo, Reema Bhagat, Neyaz Ahmad, Swetav Suman, Nalini Singh

**Affiliations:** 1 General Surgery, Rajendra Institute of Medical Sciences, Ranchi, IND

**Keywords:** burst abdomen, contaminated laparotomy, emergency laparotomy, fascial closure, incisional hernia, layered closure, mass closure, surgical site infection

## Abstract

Background: Emergency midline laparotomy in a contaminated field carries a high risk of acute fascial dehiscence (burst abdomen) and of late incisional hernia. Recommendations for abdominal wall closure derive largely from elective surgery, and the optimal anatomical closure technique in the emergency contaminated setting remains undefined. This study compared continuous mass closure with continuous layered closure in this population.

Materials and methods: We conducted a hospital-based prospective observational cohort study of 340 consecutive adults undergoing emergency midline laparotomy for contaminated intra-abdominal pathology. Patients were allocated to continuous mass closure (n = 172) or continuous layered closure (n = 168) according to the surgical unit to which they were admitted, each unit practicing one technique as its routine. All closures used 2-0 polydioxanone monofilament suture with a suture length to wound length ratio of at least 4:1. The primary outcome was burst abdomen within 30 days. Secondary outcomes were superficial and deep surgical site infection (SSI) and seroma at 30 days, incisional hernia at six, 12, and 24 months, and operative and fascial closure times. Groups were compared using the independent samples t-test or Mann-Whitney U test, the chi-square or Fisher’s exact test, multivariable logistic regression, Kaplan-Meier estimation with the log-rank test, and Cox proportional hazards regression.

Results: Baseline characteristics did not differ between groups. Burst abdomen occurred in 10 of 168 layered closure patients (6.0%) and nine of 172 mass closure patients (5.2%), a difference that was not statistically significant (relative risk 1.14, 95% CI 0.47-2.73; p = 0.958); the study had low power for this endpoint at the observed event rate. Seroma was less frequent after mass closure (14/172, 8.1% vs 27/168, 16.1%; p = 0.038). Mass closure was associated with a shorter total operative time (75.77 ± 12.27 vs 95.33 ± 15.92 minutes; p < 0.001) and a shorter fascial closure time (14.80 ± 3.81 vs 24.77 ± 5.29 minutes; p < 0.001). Cumulative incisional hernia at 24 months was 16/172 (9.3%) after mass closure and 28/168 (16.7%) after layered closure. Hernia-free survival differed between groups on log-rank testing (chi-square = 4.235; p = 0.040), and layered closure remained associated with hernia development after adjustment (adjusted hazard ratio 1.860, 95% CI 1.003-3.447; p = 0.049).

Conclusions: In this cohort, continuous mass closure was associated with fewer seromas, shorter operative and closure times, and longer hernia-free survival over 24 months than layered closure, with no difference detected in burst abdomen at 30 days. Because allocation was determined by admitting unit rather than by randomization, these findings describe association rather than causation and require confirmation in a randomized trial.

## Introduction

The vertical midline incision is the standard approach for emergency abdominal surgery, providing rapid and unrestricted access to the whole peritoneal cavity. It is also the incision most likely to fail. Acute fascial dehiscence and late incisional hernia are the two principal modes of that failure. Incisional hernia is the commonest late complication of any abdominal incision and can cause pain, bowel obstruction, incarceration, and the need for reoperation [1]. Burst abdomen is the more immediate danger, with mortality reported to be as high as 45% [2].

Recommendations for abdominal wall closure are well developed for elective surgery. The European and American Hernia Societies advise a continuous small-bites technique using slowly absorbable monofilament suture for elective midline laparotomy [3-8]. The evidence base for the emergency setting is considerably weaker. The 2023 World Society of Emergency Surgery ECLAPTE guidelines identify this gap explicitly: slowly absorbable monofilament suture is recommended, but the optimal anatomical closure technique in emergency surgery remains uncertain [1,9].

Two anatomical approaches are in common use. Mass closure incorporates all musculoaponeurotic layers in a single continuous bite while excluding subcutaneous fat, whereas layered closure approximates each fascial component separately. A meta-analysis of abdominal wound closures reported significantly fewer hernias and fewer dehiscences after mass closure than after layered closure [10-12]. A mechanistic argument has been advanced for this difference: closing layer by layer may create interlaminar dead space, which in a contaminated field could serve as a nidus for fluid collection and bacterial proliferation. This remains a hypothesis rather than a demonstrated mechanism, and the certainty of the evidence favoring mass closure in emergency contaminated surgery is low [1].

Given the morbidity of acute fascial failure and the scarcity of prospective data from contaminated emergency fields, we compared continuous mass closure with continuous layered closure in emergency midline laparotomy. The primary objective was to determine whether the incidence of burst abdomen within 30 days differed between the two techniques. Secondary objectives were to compare the cumulative incidence of incisional hernia at six, 12, and 24 months; the incidence of superficial and deep surgical site infection (SSI) and of seroma at 30 days; and the total operative time and the time dedicated to fascial closure.

## Materials and methods

Study design and setting

This was a hospital-based prospective observational cohort study conducted in the Department of General Surgery of a tertiary care teaching hospital. Consecutive adults undergoing emergency midline laparotomy for contaminated intra-abdominal pathology were enrolled between March 1, 2023 and December 24, 2023. Each patient was followed for 24 months; the final follow-up assessment was performed on December 27, 2025.

Ethical approval and consent

The study protocol was reviewed and approved by the Institutional Ethics Committee of Rajendra Institute of Medical Sciences, approval number ECR/769/INST/JH/2015/RR-24, dated 20.02.23. Written informed consent was obtained from all participants.

Participants

Patients aged 18 to 65 years of either sex undergoing emergency midline laparotomy in a contaminated field were eligible for inclusion.

Patients were excluded if massive visceral edema mandated a planned open abdomen or delayed primary closure, for example severe abdominal compartment syndrome requiring a Bogotá bag, dynamic retention sutures, or temporary negative pressure wound therapy; if they were receiving chronic high-dose immunosuppressive therapy or long-term corticosteroids; if they had a pre-existing incisional hernia or were undergoing relaparotomy through a heavily scarred previous midline incision; or if profound refractory hemodynamic instability precluded the safe completion of a primary fascial closure.

Allocation and sampling

Consecutive sampling was used. Allocation of patients to the mass closure or the layered closure cohort was determined by the surgical unit to which the patient was admitted, each unit practicing the respective closure technique as its routine. Admission to a unit followed the departmental emergency rota and was independent of patient characteristics.

Sample size

The sample size was calculated for the comparison of two independent proportions using the formula described by Sakpal [13]:

$n = \frac{(z_{\alpha/2} + z_{\beta})^{2}\,\{p_{1}(1-p_{1}) + p_{2}(1-p_{2})\}}{(p_{1}-p_{2})^{2}}$

where n is the required sample size per group, p1 is the incidence of burst abdomen in the layered closure group, p2 is the anticipated incidence in the mass closure group, z(alpha/2) is 1.96 for a two-sided significance level of 5%, and z(beta) is 0.84 for 80% power. Taking p1 as 39% and p2 as 24%, the calculated requirement was 135 patients per group. Allowing for 20% attrition, the minimum required sample was 169 patients per group, or 338 in total.

Surgical technique and data collection

Preoperative variables recorded were age, sex, body mass index (BMI), active smoking status, comorbidity, hemoglobin, serum albumin, and the Acute Physiology and Chronic Health Evaluation (APACHE) II score [14]. Intraoperative variables recorded were the primary surgical pathology, total operative time, fascial closure time, suture length, wound length, and the calculated suture length to wound length (SL:WL) ratio. Data were entered prospectively through a structured electronic data collection form.

All closures used 2-0 polydioxanone monofilament suture on a half-circle needle. Bites were placed 5 to 8 mm from the cut edge of the fascia and 5 mm apart along the incision line. The operating surgeon measured the total length of the midline fascial wound before closure and the length of residual discarded suture material after closure in order to calculate the SL:WL ratio; a ratio of at least 4:1 was required for the closure to be regarded as protocol compliant [5,15]. After completion of the fascial closure, the exposed subcutaneous tissue was irrigated with copious warm saline. All patients received standard prophylactic antibiotics.

Outcomes and follow-up

The primary outcome was burst abdomen within 30 days of surgery. Secondary outcomes were superficial SSI, deep SSI, and seroma within 30 days; cumulative incisional hernia at six, 12, and 24 months; total operative time; and fascial closure time. Emergency reoperation within 30 days was recorded as an additional safety outcome.

Operational definitions

Mass Closure

Mass closure is a continuous, single-layer closure incorporating the linea alba or anterior and posterior rectus sheaths and muscle that explicitly excludes the skin and subcutaneous tissue.

Layered Closure

Layered closure is a continuous closure performed in discrete anatomical layers (e.g., posterior sheath/fascia transversalis as one layer, followed by anterior sheath as a second distinct layer). 

Burst Abdomen

Burst abdomen refers to complete or partial separation of the continuous musculoaponeurotic layers within 30 days postoperatively, requiring clinical intervention or emergency re-operation.

Incisional Hernia

Incisional hernia is any abdominal wall gap with or without a bulge in the area of a postoperative scar area perceptible by clinical examination or targeted imaging, assessed at six, 12 and 24 months.

Wounds were assessed daily by the treating team throughout the inpatient stay and again at a documented outpatient review at 30 days. Pus culture was obtained from every wound with superficial or deep SSI, and antibiotic therapy was adjusted accordingly. Patients were then followed in a longitudinal registry with scheduled clinical assessment at six, 12, and 24 months, conducted at outpatient visits and supplemented by telephone contact. Targeted abdominal ultrasonography or computed tomography was performed at the 24-month assessment to confirm the diagnosis of incisional hernia.

Statistical analysis

Data were tabulated in Microsoft Excel (Microsoft Corporation, Redmond, WA, USA) and analyzed using IBM SPSS Statistics version 26 (IBM Corp., Armonk, NY, USA). Continuous variables are reported as mean ± standard deviation (SD) with median and interquartile range (IQR); categorical variables are reported as counts with percentages. Distributional normality was assessed using the Shapiro-Wilk test. Continuous variables were compared using the independent samples t-test where both groups were normally distributed and the Mann-Whitney U test otherwise. Categorical variables were compared using the Pearson chi-square test with Yates continuity correction, or Fisher’s exact test where any expected cell count was below five. Relative risks (RR) are reported with 95% confidence intervals (CI).

Multivariable logistic regression was used for the primary outcome of burst abdomen at 30 days, with closure technique, age, BMI, preoperative hemoglobin, serum albumin, APACHE II score, symptom duration, primary pathology, and hypertension entered as covariates; results are reported as adjusted odds ratios (aOR) with 95% CI. Comorbidity was entered as hypertension versus no hypertension, so patients with diabetes mellitus were included in the reference category. Hernia-free survival was estimated using the Kaplan-Meier method, taking as the event time the scheduled assessment at which hernia was first detected, and was compared between groups using the log-rank test. A Cox proportional hazards model adjusted for closure technique, age, BMI, preoperative hemoglobin, serum albumin, APACHE II score, symptom duration, and primary pathology was fitted to estimate the adjusted hazard ratio (aHR) for hernia development. A two-sided p-value below 0.05 was considered statistically significant.

## Results

Between March 1 and December 24, 2023, 340 consecutive patients undergoing emergency midline laparotomy for contaminated abdominal pathology satisfied the eligibility criteria and were enrolled. Of these, 168 underwent continuous layered fascial closure and 172 underwent continuous mass closure. All 340 patients completed the scheduled 24-month follow-up. No patient died during the study period and no patient was lost to follow-up.

Baseline characteristics

The demographic, anthropometric, and preoperative clinical profiles of both cohorts are presented in Table 1. The two groups were comparable across all measured baseline parameters, with no significant difference for any variable. Mean age was 39.73 ± 12.26 years in the layered closure group and 39.39 ± 12.13 years in the mass closure group. Males predominated in both cohorts, comprising 108/168 (64.3%) of the layered group and 110/172 (64.0%) of the mass closure group. Mean BMI was 19.67 ± 3.12 kg/m² and 19.50 ± 3.12 kg/m² respectively. Active smokers comprised 56/168 (33.3%) and 56/172 (32.6%). Most patients in both cohorts had no recorded comorbidity; hypertension was the commonest comorbidity, followed by diabetes mellitus. Preoperative hemoglobin values were consistent with an anemic population, and serum albumin was below 3.5 g/dL in 224/340 patients (65.9%). Mean APACHE II score was 11.61 ± 2.36 and 11.51 ± 2.15, indicating moderate physiological derangement of broadly equal severity. Duodenal perforation was the predominant operative diagnosis in both groups.

**Table 1:** Baseline demographic, anthropometric, and preoperative characteristics of study patients by closure group Continuous variables are expressed as mean ± SD (median; IQR) and categorical variables as n (%). Chi-square tests incorporate the Yates continuity correction. BMI = body mass index; APACHE II = Acute Physiology and Chronic Health Evaluation II [14]; SD = standard deviation; IQR = interquartile range. *p < 0.05 denotes statistical significance.

Variable	Layered (n = 168)	Mass (n = 172)	Test	p-value
Continuous variables, mean ± SD (median; IQR)				
Age (years)	39.73 ± 12.26 (39.50; 31.00-47.25)	39.39 ± 12.13 (40.00; 30.00-49.00)	Mann-Whitney U = 14471.5	0.98
Symptom duration (days)	2.44 ± 1.14 (2.00; 1.00-3.00)	2.43 ± 1.12 (2.00; 1.00-3.00)	Mann-Whitney U = 14514.0	0.94
BMI (kg/m²)	19.67 ± 3.12 (19.65; 17.10-21.70)	19.50 ± 3.12 (19.45; 16.90-21.73)	Mann-Whitney U = 15027.5	0.523
Preoperative hemoglobin (g/dL)	9.79 ± 1.60 (9.70; 8.70-10.72)	9.76 ± 1.43 (9.60; 8.78-10.70)	Student's t = 0.179	0.858
Serum albumin (g/dL)	3.12 ± 0.73 (3.10; 2.50-3.73)	3.16 ± 0.71 (3.10; 2.50-3.80)	Mann-Whitney U = 13950.0	0.583
APACHE II score	11.61 ± 2.36 (12.00; 9.00-14.00)	11.51 ± 2.15 (12.00; 10.00-13.00)	Mann-Whitney U = 14891.0	0.622
Categorical variables, n (%)				
Sex, male	108 (64.3%)	110 (64.0%)	Chi-square = 0.000	1
Active smoker	56 (33.3%)	56 (32.6%)	Chi-square = 0.001	0.971
Comorbidity			Chi-square = 1.920, df = 2	0.383
None	120 (71.4%)	131 (76.2%)	-	-
Hypertension-	28 (16.7%)	28 (16.3%)	-	-
Diabetes mellitus	20 (11.9%)	13 (7.6%)	-	-
Primary pathology			Chi-square = 0.826, df = 2	0.662
Duodenal perforation	91 (54.2%)	90 (52.3%)	-	-
Appendicular perforation	39 (23.2%)	47 (27.3%)	-	-
Colonic ischemia	38 (22.6%)	35 (20.3%)	-	-

Intraoperative parameters

Intraoperative parameters are detailed in Table 2. Mass closure was associated with a shorter total operative time than layered closure (75.77 ± 12.27 vs 95.33 ± 15.92 minutes; t = 12.705, p < 0.001), a mean reduction of 19.6 minutes. The time dedicated exclusively to fascial closure was likewise shorter in the mass closure group (14.80 ± 3.81 vs 24.77 ± 5.29 minutes; U = 27055.5, p < 0.001), a mean saving of approximately 10 minutes. Wound length, suture length, and SL:WL ratio did not differ between the groups. An SL:WL ratio of at least 4:1 was achieved in all 340 closures, confirming adherence to the protocol throughout the study period.

**Table 2:** Intraoperative parameters stratified by closure technique Continuous variables are expressed as mean ± SD (median; IQR). SL:WL = suture length to wound length ratio. *p < 0.05 denotes statistical significance.

Variable	Layered (n = 168)	Mass (n = 172)	Test	p-value
Total operative time (min)	95.33 ± 15.92 (94.00; 85.00-106.00)	75.77 ± 12.27 (76.00; 66.00-84.00)	Student's t = 12.705	< 0.001*
Fascial closure time (min)	24.77 ± 5.29 (24.00; 21.00-28.00)	14.80 ± 3.81 (15.00; 12.00-17.00)	Mann-Whitney U = 27055.5	< 0.001*
Wound length (cm)	18.91 ± 1.74 (18.90; 17.40-20.40)	19.19 ± 1.70 (19.10; 17.88-20.80)	Mann-Whitney U = 13128.5	0.145
Suture length (cm)	80.15 ± 7.62 (80.50; 73.65-86.90)	81.68 ± 8.09 (80.75; 75.38-88.28)	Mann-Whitney U = 12978.0	0.105
SL:WL ratio	4.24 ± 0.15 (4.23; 4.11-4.37)	4.25 ± 0.15 (4.26; 4.12-4.37)	Mann-Whitney U = 13515.5	0.304
SL:WL ratio of at least 4:1 achieved, n/N (%)	168/168 (100%)	172/172 (100%)	Not applicable	Not applicable

Thirty-day outcomes

Primary and secondary outcomes at 30 days are presented in Table 3. Burst abdomen, the primary outcome, occurred in 10/168 layered closure patients (6.0%) and 9/172 mass closure patients (5.2%), giving a relative risk of 1.14 (95% CI 0.47-2.73; p = 0.958). At the observed event rate the study had approximately 6% power to detect a difference of this magnitude, so this comparison is uninformative rather than evidence that the two techniques are equivalent for this endpoint. Among patients who developed burst abdomen, the interval to fascial dehiscence was similar in the two groups.

**Table 3:** Thirty-day postoperative outcomes stratified by closure technique Chi-square tests incorporate the Yates continuity correction; Fisher’s exact test was used where any expected cell count was below five. Days to burst abdomen are expressed as mean ± SD among patients with burst abdomen only. RR = relative risk; CI = confidence interval; SSI = surgical site infection. *p < 0.05 denotes statistical significance.

Outcome	Layered n/N (%)	Mass n/N (%)	RR (95% CI)	Test	p-value
Primary outcome					
Burst abdomen	10/168 (6.0%)	9/172 (5.2%)	1.14 (0.47-2.73)	Chi-square = 0.003	0.958
Secondary outcomes: wound complications					
Superficial SSI	26/168 (15.5%)	23/172 (13.4%)	1.16 (0.69-1.95)	Chi-square = 0.158	0.691
Deep SSI	18/168 (10.7%)	9/172 (5.2%)	2.05 (0.95-4.43)	Chi-square = 2.784	0.095
Seroma formation	27/168 (16.1%)	14/172 (8.1%)	1.97 (1.07-3.63)	Chi-square = 4.322	0.038*
Pus culture obtained	40/168 (23.8%)	31/172 (18.0%)	1.32 (0.87-2.01)	Chi-square = 1.390	0.238
Additional safety outcome					
Emergency reoperation	2/168 (1.2%)	6/172 (3.5%)	0.34 (0.07-1.67)	Fisher's exact	0.283
Days to burst abdomen, burst cases only	7.90 ± 1.52 (n = 10)	7.78 ± 1.64 (n = 9)	Not applicable	Mann-Whitney U = 45.5	1

Superficial SSI occurred in 26/168 (15.5%) layered and 23/172 (13.4%) mass closure patients (RR 1.16, 95% CI 0.69-1.95; p = 0.691). Deep SSI occurred in 18/168 (10.7%) and 9/172 (5.2%) patients respectively (RR 2.05, 95% CI 0.95-4.43; p = 0.095). Seroma was significantly less frequent after mass closure, occurring in 14/172 patients (8.1%) compared with 27/168 (16.1%) after layered closure (RR 1.97, 95% CI 1.07-3.63; p = 0.038). Pus culture was obtained from every patient with superficial or deep SSI, corresponding to 40/168 (23.8%) layered and 31/172 (18.0%) mass closure patients.

Emergency reoperation within 30 days was performed in 2/168 layered (1.2%) and 6/172 mass closure patients (3.5%; RR 0.34, 95% CI 0.07-1.67; p = 0.283). All reoperations were performed in patients who had developed burst abdomen: two of 10 dehiscences in the layered group and six of nine in the mass closure group were managed operatively, the remainder conservatively. This difference in operative management of dehiscence did not reach statistical significance (p = 0.070) and its interpretation is limited by the small number of events.

Adjusted analysis of burst abdomen

On multivariable logistic regression for the primary outcome of burst abdomen at 30 days (Table 4), layered closure carried an adjusted odds ratio of 1.242 (95% CI 0.482-3.202; p = 0.654) relative to mass closure. No covariate was independently associated with burst abdomen. With only 19 events in total, the model was fitted on fewer than two events per covariate, and the adjusted estimates should be regarded as imprecise.

**Table 4:** Multivariable logistic regression analysis for acute burst abdomen at 30 days Reference categories: closure technique = mass closure; primary pathology = appendicular perforation; comorbidity = no hypertension. aOR = adjusted odds ratio; CI = confidence interval; BMI = body mass index; APACHE II = Acute Physiology and Chronic Health Evaluation II [14]. *p < 0.05 denotes statistical significance.

Variable	aOR	95% CI	p-value
Closure technique, layered vs mass	1.242	0.482-3.202	0.654
Age, per year	0.993	0.953-1.035	0.743
BMI, per kg/m²	0.868	0.733-1.028	0.101
Preoperative hemoglobin, per g/dL	0.892	0.647-1.230	0.486
Serum albumin, per g/dL	1.211	0.623-2.353	0.572
APACHE II score, per point	0.874	0.701-1.088	0.227
Symptom duration, per day	1.095	0.720-1.663	0.672
Colonic ischemia vs appendicular perforation	1.006	0.249-4.067	0.993
Duodenal perforation vs appendicular perforation	0.945	0.305-2.923	0.922
Hypertension vs no hypertension	0.328	0.042-2.562	0.288

Incisional hernia and hernia-free survival

Cumulative incisional hernia incidence and Kaplan-Meier hernia-free survival estimates at six, 12, and 24 months are presented in Table 5. Hernia was detected in 7/168 layered (4.2%) and 2/172 mass closure patients (1.2%) at six months (RR 3.58, 95% CI 0.76-17.00; p = 0.101), in 17/168 (10.1%) and 8/172 (4.7%) at 12 months (RR 2.18, 95% CI 0.96-4.91; p = 0.085), and in 28/168 (16.7%) and 16/172 (9.3%) at 24 months (RR 1.79, 95% CI 1.01-3.19; p = 0.063).

**Table 5:** Cumulative incisional hernia incidence and Kaplan-Meier hernia-free survival at six, 12, and 24 months Kaplan-Meier hernia-free survival is expressed as estimated probability (%). The log-rank test compares overall hernia-free survival curves between groups. Chi-square tests incorporate the Yates continuity correction; Fisher’s exact test was used where any expected cell count was below five. RR = relative risk; CI = confidence interval. *p < 0.05 denotes statistical significance.

Follow-up time point	Layered n/N (%)	Mass n/N (%)	RR (95% CI)	Test	p-value
Cumulative incisional hernia					
6 months	7/168 (4.2%)	2/172 (1.2%)	3.58 (0.76-17.00)	Fisher's exact	0.101
12 months	17/168 (10.1%)	8/172 (4.7%)	2.18 (0.96-4.91)	Chi-square = 2.970	0.085
24 months	28/168 (16.7%)	16/172 (9.3%)	1.79 (1.01-3.19)	Chi-square = 3.464	0.063
Kaplan-Meier hernia-free survival, %					
6 months	0.958	0.988	Not applicable	Not applicable	Not applicable
12 months	0.899	0.953	Not applicable	Not applicable	Not applicable
24 months	0.833	0.907	Not applicable	Not applicable	Not applicable
Overall hernia-free survival	Not applicable	Not applicable	Not applicable	Log-rank chi-square = 4.235, df = 1	0.040*

Kaplan-Meier analysis (Figure 1) showed consistently higher hernia-free survival in the mass closure cohort throughout the follow-up period. At 24 months the estimated hernia-free survival probability was 90.7% in the mass closure group and 83.3% in the layered closure group. The two curves separated progressively from the six-month assessment onward, and the difference in overall hernia-free survival was statistically significant on log-rank testing (chi-square = 4.235, p = 0.040). The comparison of cumulative incidence at 24 months alone did not reach significance (p = 0.063); the log-rank test attains significance because it uses the timing of hernia detection across the whole follow-up period rather than the proportion affected at a single time point. The two results should therefore be read together rather than as one confirming the other.

**Figure 1:**
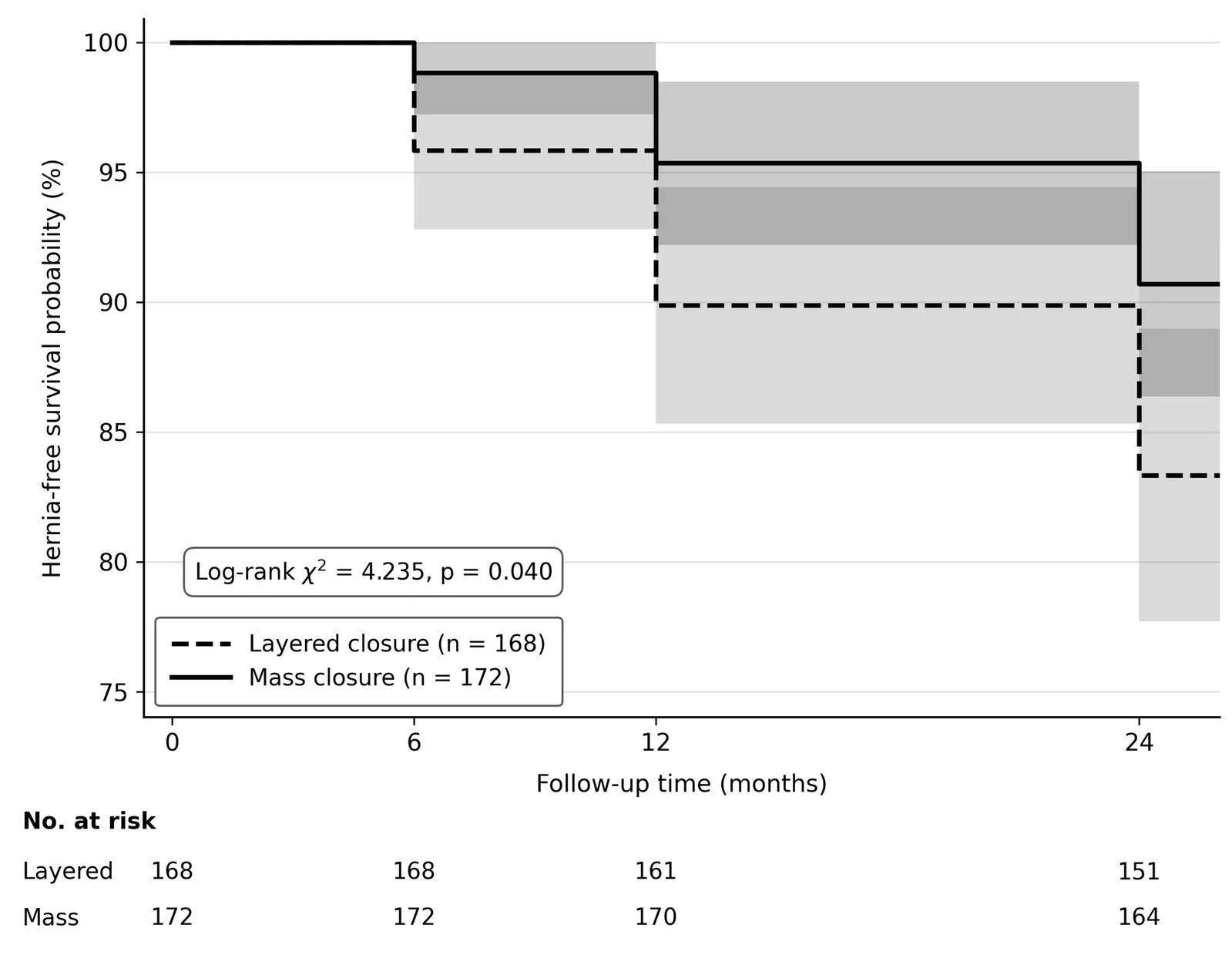
Kaplan-Meier curves of hernia-free survival at six, 12, and 24 months, stratified by fascial closure technique Kaplan-Meier hernia-free survival curves for the layered closure group (n = 168, dashed line) and the mass closure group (n = 172, solid line). Shaded bands represent 95% confidence intervals calculated by Greenwood's formula. The number-at-risk table shows the number of patients remaining hernia-free and under observation immediately before each assessment. There was no censoring: all 340 patients completed 24 months of follow-up. Hernia status was ascertained at three scheduled assessments rather than continuously, so event times are interval-detected. Log-rank chi-square = 4.235, p = 0.040.

A Cox proportional hazards model was fitted to determine the adjusted hazard of incisional hernia development (Table 6). Layered closure was associated with an adjusted hazard ratio of 1.860 (95% CI 1.003-3.447; p = 0.049) for incisional hernia over the 24-month follow-up period, corresponding to an 86% higher hazard relative to mass closure after adjustment for the measured covariates. No other covariate reached statistical significance. The lower confidence limit lies close to unity and the model was fitted on 44 events for nine covariates, so this estimate is statistically fragile.

**Table 6:** Multivariable Cox proportional hazards regression analysis for incisional hernia development at 24 months Reference categories: closure technique = mass closure; primary pathology = appendicular perforation. aHR = adjusted hazard ratio; CI = confidence interval; BMI = body mass index; APACHE II = Acute Physiology and Chronic Health Evaluation II [14]. *p < 0.05 denotes statistical significance.

Variable	aHR	95% CI	p-value
Closure technique, layered vs mass	1.86	1.003-3.447	0.049*
Age, per year	1.021	0.996-1.047	0.1
BMI, per kg/m²	0.957	0.865-1.058	0.391
Preoperative hemoglobin, per g/dL	1.114	0.920-1.348	0.268
Serum albumin, per g/dL	0.776	0.505-1.192	0.247
APACHE II score, per point	0.956	0.836-1.093	0.51
Symptom duration, per day	0.909	0.695-1.188	0.484
Colonic ischemia vs appendicular perforation	0.846	0.352-2.035	0.709
Duodenal perforation vs appendicular perforation	0.933	0.462-1.882	0.846

## Discussion

In this prospective cohort of 340 patients undergoing emergency midline laparotomy in a contaminated field, continuous mass closure and continuous layered closure produced similar rates of acute fascial dehiscence at 30 days but differed in three respects: mass closure was faster, was followed by fewer seromas, and was associated with longer hernia-free survival over 24 months. The divergence between the techniques was therefore temporal. They were indistinguishable in the early postoperative period and separated only over the subsequent two years.

Our early results are consistent with the available randomized evidence [9,16-18]. The CONTINT trial, a multicenter randomized controlled trial comparing continuous all-layer suture with interrupted closure after primary emergency midline laparotomy for a suspected intra-abdominal septic focus, found no difference between the techniques in its composite primary endpoint of burst abdomen within 30 days or incisional hernia within 12 months [17]. Peponis et al., who randomized 136 patients undergoing emergent laparotomy to interrupted or continuous fascial closure, likewise found no significant difference in incisional hernia and reported that closure took significantly longer with the interrupted technique [9,18]. Our finding of a mean saving of approximately 10 minutes in fascial closure time with mass closure is consistent with that observation. Neither trial extended surveillance to 24 months, which may explain why neither detected a late divergence of the kind seen here.

Comparative studies of modified continuous techniques in this setting have reported findings in the same direction. Aghara et al. compared modified Smead-Jones closure with conventional continuous closure in 100 patients undergoing emergency midline laparotomy and reported lower rates of wound infection and wound dehiscence with the modified technique [19]. Dhamnaskar et al., comparing modified continuous Smead-Jones closure with interrupted closure for contaminated midline laparotomy wounds, reported shorter operative times and a shorter requirement for suture material with the continuous technique, together with a lower but not statistically significant rate of wound dehiscence [20]. The mechanism proposed in both reports, namely a more even distribution of tension across the fascial edges by a continuous suture, would apply equally to continuous mass closure and offers a plausible explanation for the late benefit observed in our cohort.

The reduction in seroma after mass closure is the finding most directly attributable to the anatomical difference between the two techniques. Layered closure leaves a potential space between the separately approximated fascial layers, and in a contaminated field such a space may accumulate fluid. Whether this also accounts for the numerically lower rate of deep SSI after mass closure cannot be determined from these data, since that comparison did not reach statistical significance. Uncertainty about the optimal management of heavily contaminated abdominal wounds persists more broadly [9,11]; Venkateswaran et al. compared primary with delayed primary closure of class III and IV surgical wounds following emergency laparotomy in a comparable setting [21]. Our observation that seroma and deep SSI moved in the same direction is compatible with, but does not establish, a shared mechanism.

Limitations

Several limitations should be considered. Allocation was determined by the admitting surgical unit rather than by randomization. Although admission followed the departmental emergency rota and was independent of patient characteristics, and although the two cohorts were comparable across all 11 measured baseline variables, unit-based allocation means that the two techniques were not delivered by the same surgeons. Any systematic difference between units in operative experience, perioperative care, or threshold for reoperation would be indistinguishable from an effect of the closure technique itself. Comparability across measured variables does not exclude confounding by unmeasured factors, and neither surgeons nor outcome assessors were blinded. The study was also conducted at a single tertiary care centre, so the case mix, surgical training, and perioperative practice reflect one institution and may not generalise to other settings. Nutritional status was captured only as serum albumin and body mass index. Both were comparable between groups at baseline and were entered as covariates in the logistic and Cox models. Perioperative nutritional support was not standardised by protocol, and the route, timing, and adequacy of nutritional supplementation were not recorded. Given that serum albumin was below 3.5 g/dL in 224 of 340 patients (65.9%), residual confounding by nutritional status cannot be excluded, and any systematic difference between the two units in perioperative nutritional practice would be indistinguishable from an effect of the closure technique.

The study was substantially underpowered for its primary outcome. The sample size calculation assumed an incidence of burst abdomen of 39% in the control group, whereas the observed incidence was 6.0%. At that event rate the study had approximately 6% power for the observed difference, and the absence of a significant difference in burst abdomen must not be read as evidence that the two techniques are equivalent for this endpoint.

Both regression models were fitted on few events relative to the number of covariates, with 19 events for 10 covariates in the logistic model and 44 events for nine covariates in the Cox model, so the adjusted estimates are imprecise and may be overfitted. Comorbidity was entered in the logistic model as hypertension versus no hypertension, so patients with diabetes mellitus were included in the reference category and diabetes was not modelled as a separate level; the Cox model did not include comorbidity at all. The proportional hazards assumption was not formally tested. Multiple comparisons were made without adjustment for multiplicity, and the lower confidence limit of the 24-month adjusted hazard ratio is 1.003, so a small change in the number of events would alter its statistical significance.

Hernia status was ascertained at three scheduled assessments rather than continuously, so the survival analysis rests on interval-detected rather than exactly observed event times, and a hernia arising between assessments is assigned to the next scheduled interval. Imaging was performed at the 24-month assessment only; hernias at six and 12 months were identified clinically, so subclinical hernias present at those earlier time points may have been missed.

Finally, no patient died and no patient was lost to follow-up over the 24-month period. This reflects an intensive follow-up protocol combining scheduled outpatient review with telephone contact, but complete two-year retention with no mortality in an emergency contaminated laparotomy cohort is unusual, and readers should weigh the survival estimates with that in mind. Mortality was not a prespecified study outcome, and no assessment of cost, quality of life, or functional recovery was performed, so no conclusion can be drawn in those domains.

## Conclusions

In this prospective observational cohort of emergency midline laparotomies performed in contaminated fields, continuous mass closure and continuous layered closure produced similar rates of acute fascial dehiscence at 30 days, although the study was underpowered for this endpoint. Mass closure was associated with a shorter total operative time, a shorter fascial closure time, a lower incidence of seroma, and longer hernia-free survival over 24 months on both Kaplan-Meier and adjusted Cox analysis.

These findings suggest that the clinically relevant difference between the two techniques in this population emerges late rather than early, and that mass closure may offer an advantage for long-term abdominal wall integrity. Because allocation was determined by the admitting unit rather than by randomization, and because the adjusted hazard estimate is statistically fragile, this remains an association rather than a demonstrated causal effect. A randomized trial with surveillance extending well beyond 12 months would be required to establish whether the late divergence observed here is attributable to the closure technique itself.
